# Efficient One-Pot Synthesis of Indol-3-yl-Glycines via Uncatalyzed Friedel-Crafts Reaction in Water

**DOI:** 10.3390/molecules14031056

**Published:** 2009-03-05

**Authors:** Mehdi Ghandi, Abuzar Taheri

**Affiliations:** School of Chemistry, University College of Science, University of Tehran, Tehran, Iran

**Keywords:** Amino acid, Indol-3-yl-glycine, Glyoxalic acid, Friedel-Crafts reaction

## Abstract

The three component reaction of primary aliphatic amines, glyoxalic acid and indole or *N*-methylindole in water at ambient temperature affords indol-3-yl or *N*-methylindol-3-yl-glycine in almost quantitative yields.

## Introduction

The use of water as an environmentally benign solvent for organic synthesis has become an important research area from both the economical and synthetic point of view. The indole ring system is probably the most ubiquitous heterocycle in Nature [1]. Substituted indoles have been referred to as “privileged structures” since they are able to bind with high affinity to many receptors [2]. Indol-3-yl-glycine derivatives are one of the important non-proteinogenic amino acids for the synthesis of many biologically active compounds such as dragmacdins, hamacanthin and pemedolac [3,4,5,6,7]. Therefore, the development of new strategies to the synthesis of indol-3-yl-glycine derivatives has been the subject of considerable interest.

Owing to the importance of this class of amino acids, several procedures such as the Friedel-Crafts reaction of indole either with glyoxylate imine/iminium species or glyoxalate and amines are convenient methods for the synthesis of indol-3-yl-glycines. However, these methods in general require utilization of catalysts such as TFA, Yb(OTf)_3_, 1*H*-benzotriazole and TiCl_4_ [8,9,10,11]. Recent reports on the reaction of glyoxalic esters, amines and indole under solvent and catalyst free conditions are of fundamental interest [12,13]. Utilization of glyoxalic acid as aldehyde has been reported by Jiang *et al*., but indolyl boronic acid has been used in their approach, which does not seem to be a convenient reagent [14].

In this paper, we report the one-pot synthesis of several indol-3-yl-glycines at ambient temperature using water as solvent. The procedure is based on the uncatalyzed Friedel-Crafts condensation between indole or *N*-methylindole and various iminoacids formed *in situ* from glyoxalic acid and primary aliphatic amines.

## Results and Discussion

**Scheme 1:**
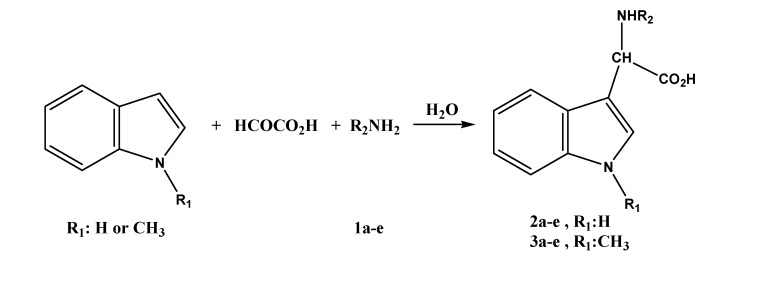
Synthesis of indol-3-yl and *N*-methylindol-3-yl-glycine.

The model three-component reaction was carried out by stirring the mixture of indole (10 mmol), glyoxalic acid (10 mmol) and butylamine (10 mmol) in water (30 mL). It was found that at least 1 h is needed for the reaction to go to completion at ambient temperature. Thus, the three-component reactions of indole or *N*-methylindole with glyoxalic acidand primary aliphatic amines **1a-e **in water for 1 h afforded the indol-3-yl (**2a-e**) or *N*-methylindol-3-yl-glycines (**3a-e**), respectively. The reaction is depicted in Scheme 1 and the results are presented in Table 1. 

**Table 1:** Yield and melting points for**2a-e** and **3a-e**.

R1	R2	Product	Yield(%)	M. P.(^ °^C)
H	CH_3_	**2a**	95	198-200
H	CH_3_CH_2_	**2b**	96	190-191
H	CH_3_(CH_2_)_2_	**2c**	95	123-125
H	CH_3_(CH_2_)_3_	**2d**	94	214-216
H	PhCH_2_	**2e**	95	200-201
CH_3_	CH_3_	**3a**	93	187-188
CH_3_	CH_3_CH_2_	**3b**	92	196-197
CH_3_	CH_3_(CH_2_)_2_	**3c**	93	197-198
CH_3_	CH_3_(CH_2_)_3_	**3d**	95	189-190
CH_3_	PhCH_2_	**3e**	96	174-176

**Figure 1:**
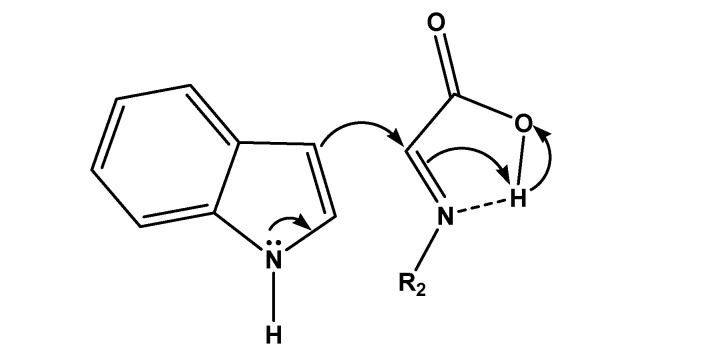
Intramolecular acid catalysis of the Friedel-Crafts reaction of indole with an iminoacid.

Attempts to carry out the reaction with secondary aliphatic amines such as pyrrolidine, piperidine and diallylamine were unsuccessful, probably due to instability of the corresponding iminium salts in water. Moreover, using aromatic amines such as aniline, and 2-aminopyridine in most of the cases, messy and sticky mixtures were formed. The high efficiency of reaction might be rationalized on the basis of Brønsted acid catalysis of the carboxylic acid, as indicated in Figure 1.

The inefficiency of aromatic amine in achieving hydrogen bonding to the acid site may be due to the weaker basic strength of the nitrogen, which is in direct conjugation with aromatic ring. Identification of the products were carried out on the basis of their spectroscopic information. For example, compound **2d** exhibited a molecular ion peak at m/z 246. The IR spectrum showed the correct stretching vibrations at 2,630 (CO_2_H) and 1,650-1,550 (C=O, C=C) cm^-1^. Its ^1^H-NMR spectrum in DMSO-d_6_ showed a triplet at 0.80 (3H*, J* = 7.2 Hz), a multiplet at 1.21 (2H), a multiplet at 1.56 (2H), two multiplets at 2.65 and 2.73 for diestereotopic CH_2_NH, a singlet at 4.55 (1H), a doublet of doublets that appears as a triplet at 6.99 (*J =* 7.4 Hz, 1H), a doublet of doublets that appears as a triplet at 7.09 (*J =* 7.5 Hz,1H), a doublet at 7.38 (*J* = 8.7 Hz, 1H), a singlet at 7.39 (1H), a doublet at 7.74 (*J* = 7.8 Hz, 1H), and a singlet at 11.33 (1H, disappeared upon addition of D_2_O). The ^13^C-NMR in DMSO-d_6_ exhibited five peaks at 14.4 to 59.25 (aliphatic carbons), eight peaks at 109.8 to 136.9 (aromatic carbons) and a peak at 169.5 (carboxylic acid C=O).

## Conclusions

In summary, a one-pot three component reaction of indole or *N*-methylindole, glyoxalic acid and primary aliphatic amines at ambient temperature in water provides an efficient and green method for the synthesis of indol-3-yl and *N*-methylindol-3-yl-glycine.

## Experimental

### General

All commercially available chemicals and reagents were purchased from the Merck Company and used without further purification. Melting points were determined with an Electrothermal model 9100 apparatus and are uncorrected. IR spectra were recorded on a Shimadzu 4300 spectrophotometer. The ^1^H- and ^13^C-NMR spectra were recorded on a Bruker DRX-500 AVANCE spectrometer. Unless otherwise specified DMSO-d_6_ was used as solvent. Chemical shifts (δ) were reported in ppm and referenced to the NMR solvent. Mass spectra of the products were obtained with a HP (Agilent technologies) 5937 Mass Selective Detector.

### General procedure for the synthesis of indol-3-yl or N-methylindol-3-yl-glycines2a-e and 3a-e

To a solution of indole or *N*-methylindole (10 mmol) and glyoxalic acid (10 mmol) in water (30 mL) was added aliphatic amine (10 mmol) and the mixture was stirred for 1 h at ambient temperature. After filtration of the precipitate formed, the solid was purified by trituration in hot methanol and then in hot ethylacetate.

*Indol-3-yl-N-methylglycine* (**2a**): White solid; IR (KBr): 3448 (NH), 3153, 2993, 2875, 2528 (CO_2_H), 1645 (C=O), 1602 cm^-1^; ^1^PH-NMR δ: 2.35 (s, 3H), 4.48 (s, 1H), 6.99 (t, *J* = 7.7 Hz, 1H), 7.08 (t, *J* = 7.9 Hz, 1H), 7.36 (s, 1H), 7.37 (d, *J* = 7.9 Hz, 1H), 7.74 (d, *J* = 7.7 Hz, 1H), 11.34 (s, 1H) ppm;^ 13^C-NMR δ: 31.9, 60.5, 109.5, 112.3, 119.5, 120.5, 122.1, 126.7, 127.2, 137.0, 169.2 ppm; MS (EI): m/z 149 (M^+^-45).

*Indol-3-yl-N-ethylglycine* (**2b**): Cream solid; IR (KBr): 3514, 3186, 2877,2763, 2592 (CO_2_H), 1625 (C=O), 1602 cm^-1^; ^1^PH-NMR δ: 1.13 (dd, 3H), 2.69 (m, 1H), 2.80 (m, 1H), 4.52 (s, 1H), 6.99 (t, *J* = 7.5 Hz, 1H), 7.08 (t, *J* = 7.7 Hz, 1H), 7.36-7.37 (bd, 2H), 7.73 (d, *J =* 7.5 Hz, 1H), 11.24 (s, 1H) ppm;^ 13^C- NMR δ: 11.9, 41.2, 58.8, 109.8, 112.3, 119.5, 120.3, 122.1, 126.4, 127.3, 136.9, 169.0 ppm; MS (EI): m/z 218 (M^+^).

*Indol-3-yl-N-propylglycine* (**2c**): Cream solid; IR (KBr): 3109, 2960, 2711, 2559 (CO_2_H), 1645 (C=O), 1600 cm^-1^; ^1^PH-NMR δ: 0.79 (t, *J*=7.4 Hz, 3H), 1.59 (m, 2H), 2.62 (m, 1H), 2.69 (m, 1H), 4.59 (s, 1H), 6.99 (t, *J =* 7.5 Hz, 1H), 7.08 (t,*J =* 7.8 Hz, 1H), 7.38 (d, *J =* 7.8 Hz, 1H), 7.4 (s, 1H), 7.73 (d, *J* = 7.5 Hz, 1H), 11.42 (s, 1H) ppm;^ 13^C-NMR δ: 11.9, 19.8, 47.9, 59.1, 109.5, 112.4, 119.5, 120.2, 122.0, 126.6, 127.3, 137.0, 169.7 ppm; MS (EI): m/z 232 (M^+^).

*Indol-3-yl-N-butylglycine* (**2d**): Light pink solid; IR (KBr): 3492 (NH), 3321, 3060,2933, 2759, 2630 (CO_2_H), 1650-1550 (C=O, C=C) cm^-1^; ^1^PH-NMR δ: 0.80 (t*, J* = 7.2 Hz, 3H), 1.21 (m, 2H), 1.56 (m, 2H), 2.65 (m, 1H), 2.73 (m, 1H), 4.55 (s, 1H), 6.99 (t,*J =* 7.5 Hz, 1H), 7.09 (t, *J =* 7.8 Hz, 1H), 7.38 (d, *J* = 7.8Hz, 1H), 7.39 (s, 1H), 7.74 (d, *J* = 7.5 Hz), 11.33 (s, 1H) ppm;^ 13^C-NMR δ: 14.4, 20.3, 28.4, 46.1, 59.2, 109.9, 112.3, 119.5, 120.3, 122.0, 126.5, 127.4, 136.9, 169.5 ppm; MS (EI): m/z 246 (M^+^).

*Indol-3-yl-N-benzylglycine* (**2e**): Dark pink solid; IR (KBr): 3373 (NH), 3213, 3109, 2991, 2493, 2629 (CO_2_H), 1650-1550 (C=O, C=C) cm^-1^; ^1^PH-NMR δ: 3.89 (AB, *J* = 13.3 Hz, 2H), 4.47 (s, 1H), 6.98 (t, *J =* 7.3 Hz, 1H), 7.09 (t,* J =* 7.6 Hz, 1H), 7.32- 7.60 (m, 7H), 7.60 (d, *J* = 7.6 Hz, 1H), 11.13 (s, 1H) ppm;^ 13^C-NMR δ: 48.8, 55.3, 112.4, 117.7, 120.5, 122.8, 124.9, 127.7, 128.9, 129.5, 129.7, 136.0, 170.1 ppm; MS (EI): m/z 280 (M^+^). 

*N-methylindol-3-yl-N-methylglycine*(**3a**): White solid; IR (KBr): 3111, 3003, 2879, 2522 (CO_2_H), 1643 (CO), 1598 cm^-1^; ^1^PH-NMR δ: 2.36 (s, 3H), 3.78 (s, 3H), 4.40 (s, 1H), 7.04 (t,* J =* 7.7 Hz, 1H), 7.17 (t,* J =* 7.8 Hz, 1H), 7.33 (s, 1H), 7.42 (d, *J* = 7.8 Hz, 1H), 7.75 (d, *J* = 7.7 Hz, 1H) ppm;^ 13^C-NMR (D_2_O + HCl) δ: 30.5, 32.6, 57.2, 100.9, 110.7, 118.1, 120.7, 122.8, 125.4, 132.3, 137.1, 170.69 ppm; MS (EI): m/z 218 (M^+^).

*N-methylindol-3-yl-N-ethylglycine*(**3b**): White solid; IR (KBr): 3109, 2979, 2680, 2534 (CO_2_H), 1650 (CO), 1596 cm^-1^; ^1^PH-NMR δ: 1.14 (t, *J* = 7.1 Hz, 3H), 2.70 (m, 1H), 2.80 (m, 1H), 3.78 (s, 3H), 4.46 (s, 1H), 7.05 (t,* J =* 7.5 Hz, 1H), 7.17 (t, *J =* 7.8 Hz, 1H), 7.35 (s, 1H), 7.42 (d, *J* = 7.8 Hz, 1H), 7.75 (d, *J* = 7.5 Hz, 1H) ppm;^ 13^C-NMR (D_2_O + HCl) δ: 10.6, 32.7, 41.0, 55.8, 101.5, 110.8, 118.2, 120.8, 122.9, 125.6, 132.0, 137.1, 170.8 ppm; MS (EI): m/z 232 (M^+^).

*N-methylindol-3-yl-N-propylglycine*(**3c**): White solid; IR (KBr): 3111, 2966, 2825, 2549 (CO_2_H), 1630 (CO), 1573 cm^-1^; ^1^PH-NMR δ: 0.43 (t,*J* = 8 Hz, 3 H), 1.23 (m, 1 H), 1.3 (m, 1H), 2.43 (m, 1H), 2.51 (m, 1H), 3.25 (s, 1 H), 6.8 (m, 2 H), 6.91 (d, *J* = 7.8 Hz, 1H), 7.12 (s, 1H), 7.28 (d,* J* = 7.6 Hz, 1H) ppm; ^13^C-NMR (D_2_O + HCl) δ: 10.5, 19.2, 32.7, 47.2, 56.0, 101.6, 110.8, 118.3, 120.8, 122.9, 125.9, 131.9, 137.1, 170.9 ppm; MS (EI): m/z 201 (M^+^-45).

*N-methylindol-3-yl-N-buthylglycine*(**3d**): Light pink solid; IR (KBr): 3111, 2934, 26940, 2549(CO_2_H), 1620 (CO), 1596 cm^-1^; ^1^PH-NMR δ: 0.83 (t, *J =* 6.5 Hz, 3H), 1.23 (m, 2H), 1.55 (m, 2H), 2.80 (m, 2H), 3.73 (s, 3H), 4.45 (s, 1H), 7.02 (m, 1H), 7.14 (m, 1H), 7.30 (s, 1H), 7.37 (d, *J* = 7.9 Hz, 1H), 7.74 (d, *J* = 7.7 Hz, 1H) ppm;^ 13^C-NMR (D_2_O + HCl) δ: 12.8, 19.3, 27.4, 32.8, 45.5, 56.0, 101.4, 110.9, 118.2, 120.8, 122.9, 125.7, 132.1, 137.1, 170.8 ppm; MS (EI): m/z 260 (M^+^).

*N-methylindol-3-yl-N-benzylglycine*(**3e**): White solid; IR (KBr): 3438, 3069, 2825, 2333 (CO_2_H), 1635 (CO), 1600 cm^-1^; ^1^PH-NMR δ: 3.75 (s, 3H), 3.88 (AB, *J=* 13.4 2H), 4.48 (s, 1H), 7.02 (t,* J =* 7.5 Hz, 1H), 7.16 (t, *J =* 7.7 Hz, 1H), 7.31- 7.42 (m, 7H), 7.61 (d, *J* = 7.5 Hz, 1H) ppm;^ 13^C-NMR δ: 33.3, 50.5, 58.1, 110.5, 119.7, 120.4, 122.2, 127.5, 128.6, 129.2, 129.9, 136.9, 137.4, 171.5 ppm; MS (EI): m/z 294 (M^+^).
